# Vaccinia-Related Kinase 1 Is Required for the Maintenance of Undifferentiated Spermatogonia in Mouse Male Germ Cells

**DOI:** 10.1371/journal.pone.0015254

**Published:** 2010-12-13

**Authors:** Yoon Ha Choi, Choon-Ho Park, Wanil Kim, Hua Ling, Aram Kang, Matthew Wook Chang, Sun-Kyoung Im, Hyun-Woo Jeong, Young-Yun Kong, Kyong-Tai Kim

**Affiliations:** 1 Division of Molecular and Life Science, Department of Life Science, Pohang University of Science and Technology (POSTECH), Pohang, Republic of Korea; 2 School of Chemical and Biomedical Engineering, Nanyang Technological University, Singapore, Singapore; 3 Department of Biological Sciences, Seoul National University, Seoul, Republic of Korea; 4 Division of Integrative Bioscience and Biotechnology, Pohang University of Science and Technology (POSTECH), Pohang, Republic of Korea; Brigham and Women's Hospital, United States of America

## Abstract

Vaccinia-related kinase 1 (VRK1) is a crucial protein kinase for mitotic regulation. VRK1 is known to play a role in germ cell development, and its deficiency results in sterility. Here we describe that VRK1 is essential for the maintenance of spermatogonial stem cells. To determine whether VRK1 plays a role in these cells, we assessed the population size of undifferentiated spermatogonia. Flow cytometry analyses showed that the number of undifferentiated spermatogonia was markedly reduced in VRK1-deficient testes. VRK1 was highly expressed in spermatogonial populations, and approximately 66% of undifferentiated spermatogonia that were sorted as an Ep-CAM^+^/c-kit^−^/alpha-6-integrin^+^ population showed a positive signal for VRK1. Undifferentiated stem cells expressing Plzf and Oct4 but not c-kit also expressed VRK1, suggesting that VRK1 is an intrinsic factor for the maintenance of spermatogonial stem cells. Microarray analyses of the global testicular transcriptome and quantitative RT-PCR of VRK1-deficient testes revealed significantly reduced expression levels of undifferentiated spermatogonial marker genes in early postnatal mice. Together, these results suggest that VRK1 is required for the proliferation and differentiation of undifferentiated spermatogonia, which are essential for spermatogenic cell maintenance.

## Introduction

Spermatogenesis is a highly productive and tightly regulated process. Multiple cell types, including somatic and germ cells, play different roles to maintain continuous sperm cell production. To sustain the germ cell population, the active proliferation of spermatogonia is required. Moreover, the proliferation rate, which is one of the highest in the body, must be well regulated. Spermatogonial stem cells (SSCs) are adult stem cells in the testes that support the germ cell pool throughout a male's life. Like other stem cells, SSCs can self-renew and differentiate into spermatozoa. These stem cells can proliferate rapidly to restore the germ cell population if that population becomes diminished (e.g., following exposure to a toxic chemical agent or irradiation). However, under normal physiological conditions, these cells divide slowly to generate both stem cells and progenitor cells [1]. Unidentified subpopulations of undifferentiated spermatogonia have stem cell capabilities. Undifferentiated spermatogonia comprise A_single_ (A_s_), A_paired_ (A_pr_), and A_aligned_ (A_al_) cells among type A spermatogonia [2], [3], [4].

Vaccinia-related kinases (VRKs) belong to the casein kinase family whose catalytic domain shares homology with the vaccinia virus gene, B1R [5]. Mammalian genomes encode three types of VRK proteins, namely VRK1, VRK2, and VRK3. VRK1 and VRK3 possess C- and N-terminal nuclear localization signals, respectively, while VRK2 has a C-terminal transmembrane domain. Interestingly, while VRK1 and VRK2 are enzymatically active, VRK3 is claimed as a pseudokinase whose substrates have not yet been identified [6]. VRK1 is highly expressed in both actively proliferating tissues, human tumor cell lines, and several tumor tissues suggesting that it plays an important role in cell cycle progression [5], [7], [8]. Recent studies have revealed several possible mechanisms by which VRK1 controls cell cycle progression. We have shown that VRK1 phosphorylates histone H3 on Thr3 and Ser10 during mitosis. Phosphorylation of the above residues is involved in important mitotic signals that induce chromatin condensation [9]. In addition, VRK1 is known to phosphorylate barrier-to-autointegration factor (BAF), which is critical for supporting both the nuclear envelope and chromatin structure. VRK1-mediated phosphorylation of BAF triggers BAF release from nuclear membrane proteins that contain LEM domains, causing disintegration of the nuclear envelope during early mitosis [10]. Furthermore, VRK1 expression is necessary for cyclin D1 expression and G1 progression [11], [12]. Mechanistically this is due to the incorporation of VRK1 to the cyclin D1 gene transcription complex where VRK1 phosphorylates cAMP response element-binding protein in Ser133, which is important for cyclin D1 induction [12]. Finally, VRK1 has also been known to phosphorylate several transcription factors, such as p53 [13], [14], ATF2 [15], and c-Jun [16], to increase transcription factor stability and activity.

Recently, many researchers have made an effort to examine the genetics of VRK1 in various organisms. A VRK1 gene-depleted mammalian model was first reported by Wiebe et al. [17]. A VRK1 gene deficiency gives rise to defects in gametogenesis, resulting in infertility in both sexes. In male mice, hypomorphism of VRK1 leads to progressive germ cell loss caused by an impairment of spermatogonial proliferation. VRK1 deficiency results in the progressive loss of spermatogonia that exhibit GCNA^+^ (germ cell nuclear antigen) and PCNA^+^ (proliferating cell nuclear antigen).

In this report, we also generated VRK1-deficient mice that have a reporter construct in the *Vrk1* intron region and asked whether a VRK1 deficiency might affect the maintenance of spermatogonial stem cells. To test our hypothesis, we quantified stem cell population in the testes of VRK1-deficient mice using flow cytometry. In addition, we also analyzed the testicular transcriptome of VRK1-deficient mice using DNA microarray and quantitative RT-PCR to investigate gene expression changes of specific markers during the first wave of spermatogenesis in postnatal mice. Here we present evidence to suggest that VRK1 is essential for the proper maintenance of undifferentiated spermatogonia.

## Results

### Expression patterns of VRK1 in germ cell populations

**Figure 1:**
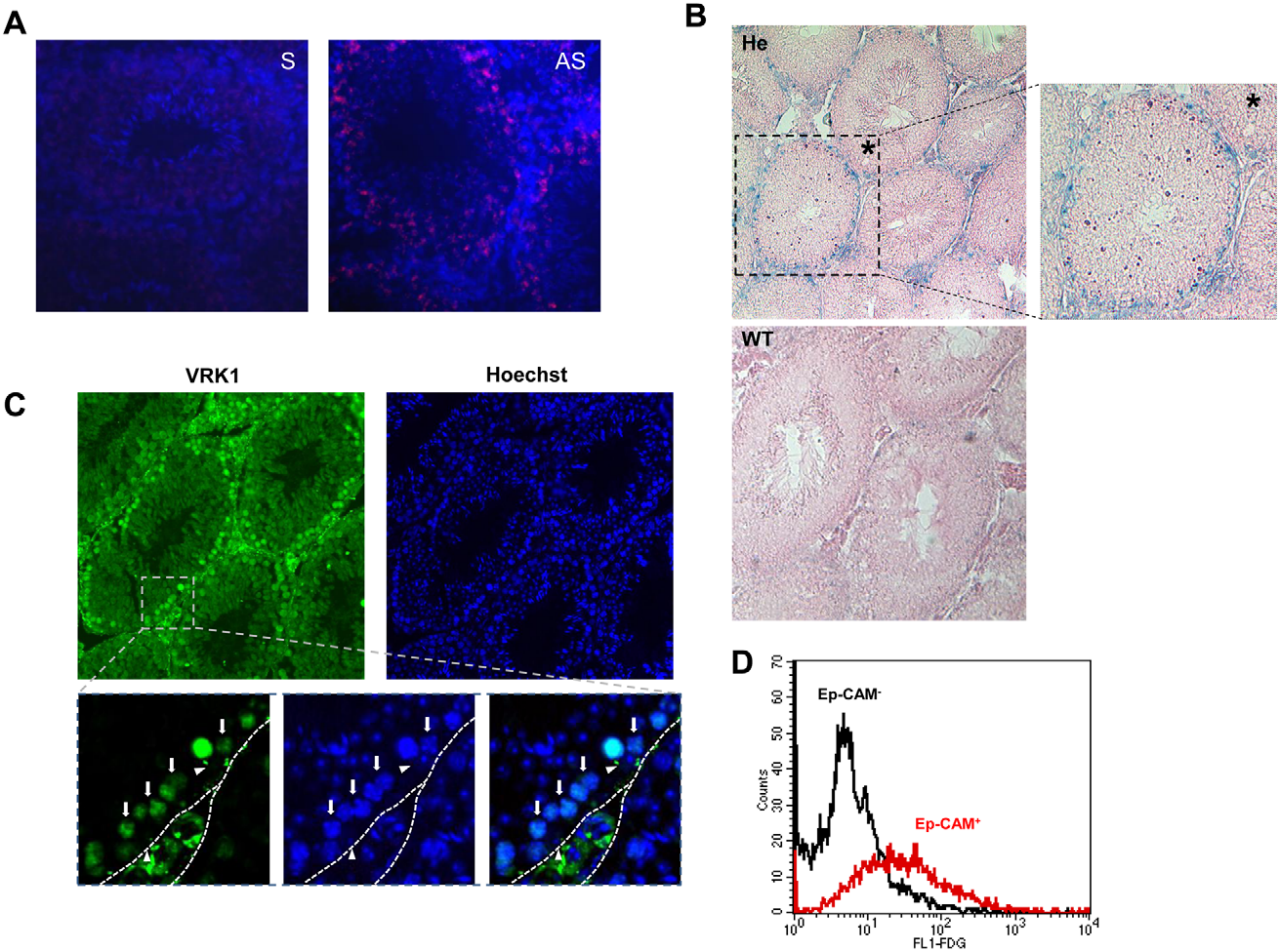
Expression of VRK1 in spermatogonial cells. (A) In situ hybridization with sense (S) or antisense (AS) probes against VRK1 mRNA (red). All testicular cells were counterstained with Hoechst 33342 (blue). (B) A section of testis from a heterozygous mouse was stained for β-galactosidase (blue) and counterstained with eosin (pink). Positive blue staining specifies the spermatogonia. He: heterozygote testis, WT: wild type testis. (C) Immunohistochemisty using a polyclonal antibody against VRK1 on a cross-section of testes isolated from an adult wild type mouse. Arrowheads and arrows in the lower panels indicate localization of Sertoli and spermatogonia, each. (D) Flow cytometry on freshly dissociated adult germ cells from a heterozygote that were positively or negatively sorted with Ep-CAM antibody. Cells were stained with FDG to identify VRK1-expressing cells.

For the genetic analysis of VRK1 in mice, we used VRK1-deficient mice as previously reported (Figure S1) [17]. This mouse contains a β-geo insertion in intron 3 of VRK1, as a result of the expression of N-terminal 72 amino acids of VRK1 fused to β-geo. As previous study revealed that this mouse is hypomorphic for VRK1, we also confirmed some of full length VRK1 was expressed in the homozygote (Figure S1) [17]. In the testis, it was confirmed that VRK1 messenger RNA was localized to the periphery of seminiferous tubules in wild-type testis but was absent centrally (Figure 1A). Histological examination of the testes from heterozygote with the β-galactosidase substrate X-gal (5-bromo-4-chloro-3-indolyl-β-D-galactoside) showed that VRK1 expression was restricted to the seminiferous tubules and was confined within the first layer of cells adjacent to the basement membrane. Therefore, we confirmed that the *Vrk1* gene is successfully trapped and expresses the β–geo fusion protein in this model system (Figure 1B).

According to a previous report, VRK1 is expressed in spermatogonia and Sertoli cells [17]. But, to date, the localization of VRK1 in mouse organs has not been confirmed with a VRK1-specific antibody. We generated anti-mouse VRK1 antibody from rabbits for further research, and its specificity was verified by Western blot analysis (Figure S2). Fifty micrograms of nucleus extracts from several organs were loaded onto an SDS/PAGE gel, and VRK1 was detected. The band of mouse VRK1 (51 kDa) was detected in thymus, spleen, and testis of wild type mice, but we could not detect any signal from the above-mentioned tissues in VRK1-deficient mice (Figure S2). According to a previous report, transcripts from VRK1 deficient mice were reduced by 80–85% compared with wild type [17]. Therefore, VRK1 protein appeared too low to detect with anti-VRK1 antibody in VRK1-deficient mice.

To test anti-VRK1 antibodies in mouse tissues, we examined testis samples from wild-type mice. VRK1 was expressed along the basal layer of the testis in which A and B type spermatogonia were aligned (Figure 1C). Although VRK1 was expressed in spermatogonia (Figure 1C, indicated by arrows), in contrast to previous report, we could not detect VRK1 protein in Sertoli cells by immunohistochemistry (Figure 1C, indicated by arrowheads)[17]. We also assessed the expression levels of VRK1 in spermatogonial populations using flow cytometry. To quantify the expression levels of VRK1 in spermatogonia, cells from VRK1-deficient mice were stained with fluorescein di-D-galactopyranoside (FDG), a substrate for β-galactosidase. As a result, the cells labeled with Ep-CAM were positive for fluorescein. These results indicated that spermatogonia specifically express VRK1 (Figure 1D).

### Defects in cell cycle progression in spermatogonia of VRK1- deficient early postnatal mice

**Figure 2:**
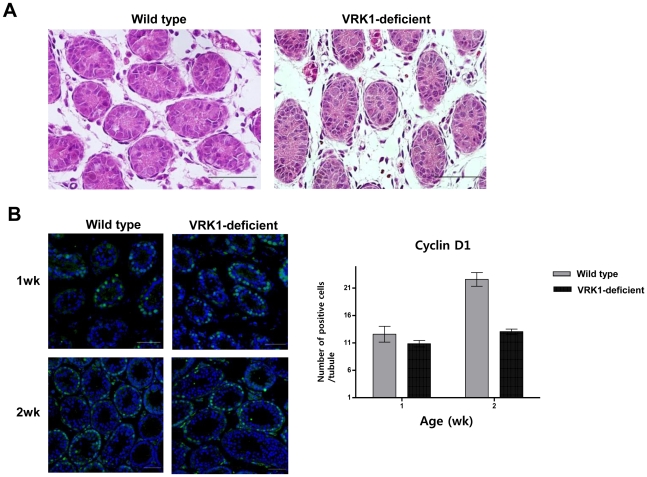
Defects in spermatogonia caused by aberrant cell proliferation in VRK1-deficient testes. (A) Histology of testes from 1-week-old VRK1-deficient. Testes from wild type and VRK1-deficient mice were stained with hematoxylin and eosin. Scale bar, 200 µm. (B) Immunohistochemistry with anti-cyclin D1 antibody for wild type testes (*n* = 3) and VRK1-deficient (*n* = 3) mice at 1 week and 2 weeks of age. Scale bar, 200 µm. More than 30 tubules were scored for each genotype. The mean value is shown with the standard error.

As degeneration of testes can be induced by a spermatogonial proliferation defect, we assessed proliferating spermatogonia in VRK1-deficient testes by immunostaining for cyclin D1, a marker of mitotically active spermatogonia (Figure 2B). Although we observed no obvious degeneration of tubules at an early age (Figure 2A), the number of proliferating spermatogonia began to decrease within 2 weeks (Figure 2B).

**Figure 3:**
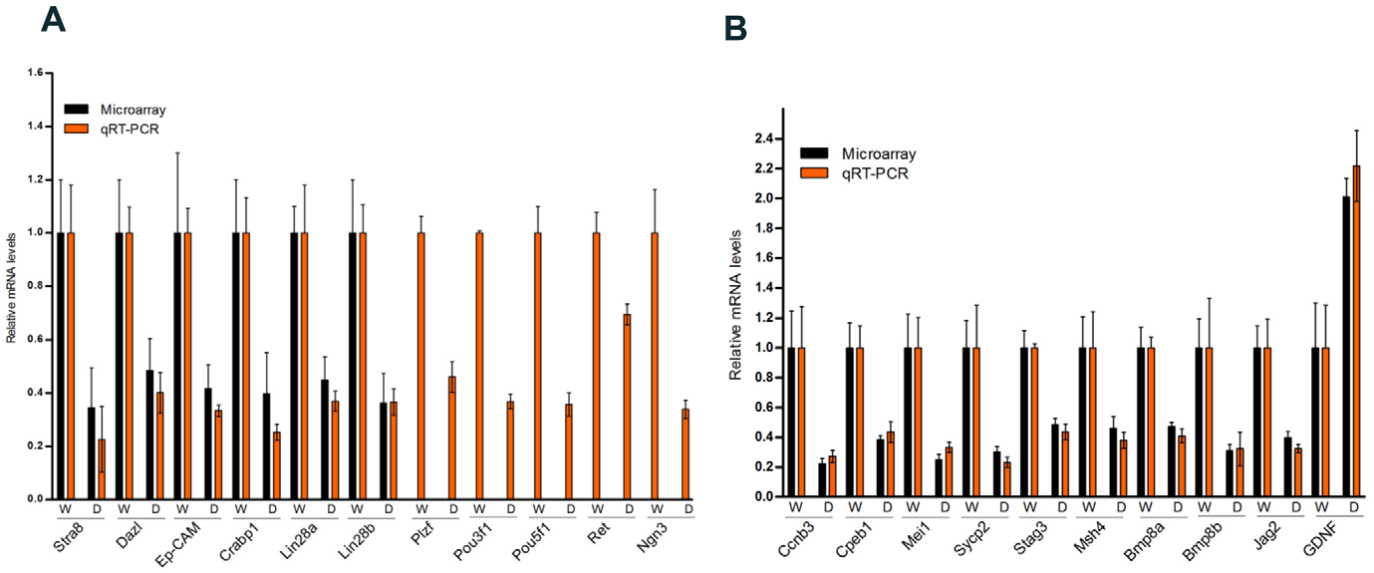
Impaired expression of genes involved in spermatogenesis at an early postnatal age. (A, B) DNA microarray and quantitative RT-PCR analysis of spermatogonial marker genes (A) or spermatogenic genes (B) in total testicular cells from postnatal day 8 of wild-type and VRK1-deficient mice. Each value of qRT-PCR was normalized to β-actin expression levels and expressed as the fold change relative to the levels detected in wild type samples, which were set equal to 1. Error bars represent the mean ± SD. Primers for qRT-PCR were presented in Table S1. W: wild type testes, D: VRK1-deficient testes.

To assess the effects of VRK1 deficiency on spermatogenesis in detail, we analyzed gene expression levels in the testis by microarray (Table 1). Testicular cells of pups (day 8) were subjected to analysis; at this age, spermatogonia actively proliferate and early types of spermatocytes appear [18]. We found that many genes related to spermatogenesis were disrupted; particularly, spermatogonia-specific transcripts (Lin28, Stra8, Dazl, Ep-CAM, and Crabp1) were downregulated. Lin28, a pluripotency factor, is expressed in pluripotent cells and undifferentiated spermatogonia [19]. Although genes expressed in undifferentiated spermatogonia were not abundantly detected by microarray analysis, we identified that several marker genes in undifferentiated spermatogonia, including Plzf, Pou3f1, Pou5f1, Ret and Ngn3 were significantly reduced by qRT-PCR (Figure 3A). Because undifferentiated spermatogonia were present at a low concentration, these genes could not be detected by microarray analysis. We confirmed the differential expression of select genes by qRT-PCR and showed an overall concordance of relative expression levels with those obtained by microarray analysis (Figure 3A and 3B). This finding indicates that VRK1 plays a critical role in the maintenance of spermatogonia during an early postnatal age.

**Table 1:** Microarray-based transcriptome analysis of testicular cells in adult wild type and VRK1-deficient mice.

**Spermatogonia**	**p-value**	**Fold change**	**Regulation**
Stra8	0.031	2.79	Down
Dazl	0.031	2.03	Down
Tacstd1 (Ep-CAM)	0.042	2.18	Down
Crabp1	0.034	2.51	Down
Lin28a	0.010	2.17	Down
Lin28b	0.002	2.85	Down
**Meiosis**	**p-value**	**Fold change**	**Regulation**
Ovol1	0.014	2.93	Down
Ccnb3	0.006	4.13	Down
Cpeb1	0.003	2.51	Down
Mei1	0.010	3.67	Down
Ccna1	0.037	2.07	Down
Sycp2	0.004	3.17	Down
Syce1	0.020	2.37	Down
Boll	0.018	2.36	Down
Smc1b	0.033	2.22	Down
Stag3	0.004	2.04	Down
Msh4	0.050	2.13	Down
Clgn	0.037	2.05	Down
Suv39h2	0.036	2.03	Down
**Spermatogenesis**	**p-value**	**Fold change**	**Regulation**
Tssk3	0.012	2.77	Down
Rnf17	0.023	2.26	Down
Mov10l1	0.017	2.25	Down
Ovol1	0.014	2.93	Down
Prok2	0.014	4.85	Down
Gal3st1	0.034	3.97	Down
Bmp8a	0.006	2.06	Down
Bmp8b	0.007	3.08	Down
Mtl5	0.013	2.43	Down
Dazl	0.031	2.03	Down
Spag16	0.004	3.35	Down
Ccna1	0.037	2.07	Down
Hmga1	0.018	2.06	Down
Boll	0.018	2.36	Down
Nlrp14	0.010	2.80	Down
Msh4	0.050	2.13	Down
Clgn	0.037	2.05	Down
Hsf2bp	0.002	3.53	Down
Dnmt3a	0.010	2.04	Down
Jag2	0.006	2.45	Down
Gdnf	0.027	2.25	Up

**Differently expressed genes involved in spermatogonia, meiosis, and spermatogenesis in VRK1-deficient testes were listed.**

Altered genes were classified into functional categories using Gene Ontology (GO) (Table S2). We found some genes that are able to globally influence spermatogenesis (Table 1 and Figure 3B). VRK1 deficiency caused a reduction in Bmp8 (bone morphogenetic protein 8) expression levels. Bmp8a and b are ubiquitously expressed in spermatogonia and primary spermatocytes before 3.5 weeks of age. Bmp8 absence results in an impairment of germ cell proliferation and differentiation [20], [21]. Glial cell line-derived neurotrophic factor (GDNF) is produced by Sertoli cells and controls the maintenance of germ line stem cells. Loss of germ cells caused by drug treatment and cryptorchid procedures results in an increase in GDNF expression [22], [23]. Interestingly, GDNF gene expression increased in VRK1-deficient testes, perhaps to repair impaired spermatogonial function. We also found that the expression levels of several genes in the meiosis category were perturbed, indicating a functional impairment of VRK1 during the first wave of the spermatogenesis.

### Expression levels of VRK1 in undifferentiated spermatogonia

**Figure 4:**
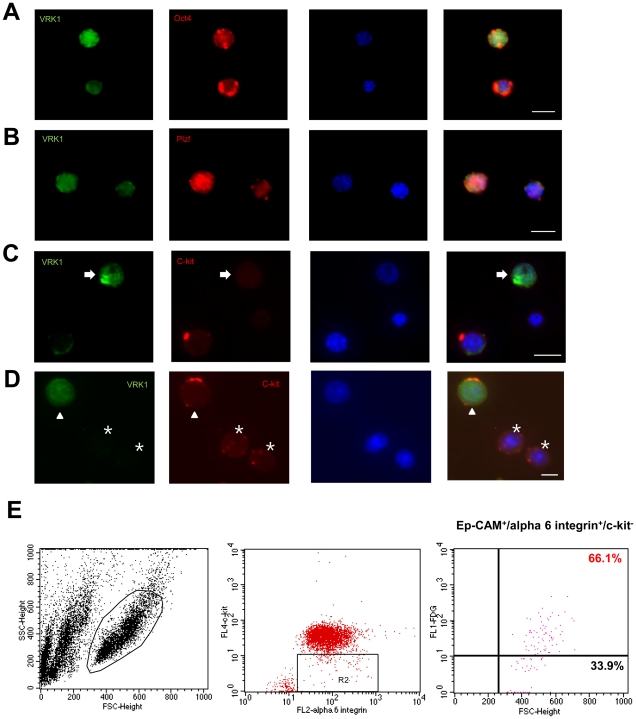
Expression of VRK1 in undifferentiated spermatogonia. (A, B) Immunocytochemistry of spermatogonia sorted by Ep-CAM antibody with VRK1 and Oct-4 (A) or Plzf (B). (C, D) Spermatogonia were stained with VRK1 and c-kit, and spermatogonia expressing VRK1 were negatively stained with c-kit (C). Heterogeneous populations stained with VRK1 were shown in c-kit-positive spermatogonia (D). (E) Flow cytometry analysis of VRK1-expressing spermatogonia that were then gated by c-kit^−^ and α6-integrin^+^ in the isolated Ep-CAM^+^ fraction of heterozygous spermatogonia. Cells were freshly stained with FDG.

Several factors influence the cell cycle progression of undifferentiated spermatogonia, including hormones, growth factors, Sertoli cells, and other germ cells. Because of the progressive degeneration and agametic phenotype of VRK1-deficient testes, we investigated whether VRK1 directly influences the function of undifferentiated spermatogonia in a cell-autonomous manner. To determine whether VRK1 is expressed in undifferentiated spermatogonia, we performed immunocytochemistry with Ep-CAM-positive spermatogonial cells sorted by MACS. We found that undifferentiated spermatogonia expressing Oct-4 and Plzf also expressed VRK1 (Figure 4A and 4B). C-kit has been detected in a broad range of type A and differentiated spermatogonia [24], [25]; undifferentiated spermatogonia lacking c-kit appeared to express VRK1 (Figure 4C, indicated by arrow). Within c-kit-positive spermatogonia, there were separate populations that either expressed VRK1 or not, meaning that VRK1 is not expressed in all differentiated spermatogonial populations (Figure 4D, indicated by arrowhead and asterisk).

To further confirm VRK1 expression levels in undifferentiated spermatogonia, we also quantified VRK1 expression levels by flow cytometry (Figure 4E). Spermatogonia were stained with Ep-CAM antibody and sorted by MACS. Cells expressing VRK1 were stained with fluorescein processed by FDG. To identify undifferentiated spermatogonial populations, cells were labeled with anti-α_6_-integrin and c-kit antibody [25]. As a result, nearly 66% of cells that were α_6_-integrin^+^ and c-kit^−^ expressed VRK1 (Figure 4E). As the data demonstrate, cells expressing makers for undifferentiated spermatogonia also expressed VRK1, suggesting that the defect in VRK1-deficient testes might be inherent for undifferentiated spermatogonia.

### Age-dependent loss of undifferentiated spermatogonia in VRK1-deficient^−^ mice

**Figure 5:**
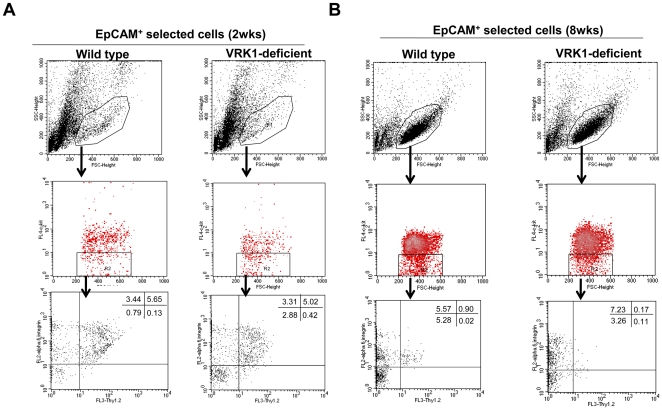
Loss of undifferentiated spermatogonia in VRK1-deficient testes. (A, B) Flow cytometric analysis of undifferentiated spermatogonia, defined as c-kit^−^, α6-integrin^+^, and Thy1^+^, in the isolated EpCAM^+^ fraction in 2- (A) and 8- (B) week-old mice.

We postulated that the gradual depletion of germ cells in aging VRK1-deficient mice was accelerated by dysfunctional undifferentiated spermatogonia. To examine this hypothesis, we also used flow cytometry to study the proportional change in undifferentiated spermatogonia in cells harvested from VRK1-deficient mice. The method published by Takubo et al. and Kubota et al. was used with few modifications to isolate undifferentiated spermatogonia [26], [27]. In purified spermatogonia, cells with high forward light scatter and low side-angle light scatter values [25] were first gated by c-kit expression and then by α_6_-integrin and Thy1.2 expression. The undifferentiated spermatogonial population, defined as c-kit^−^, α_6_-integrin^+^, and Thy1.2^+^, was reduced slightly in 2-week-old VRK1-deficient mice (Figure 5A). This change was further pronounced in 8-week-old mice as undifferentiated spermatogonial cell fractions constituted only 0.17% of the testis cell population. In wild-type testes, these cells composed 0.9% of the population (Figure 5B). Together, these data support the hypothesis that VRK1 deficiency in mouse testes disrupts undifferentiated spermatogonia, leading to a decrease in this cell population.

### Defects in the proliferation of undifferentiated spermatogonia in VRK1-deficient mice

**Figure 6:**
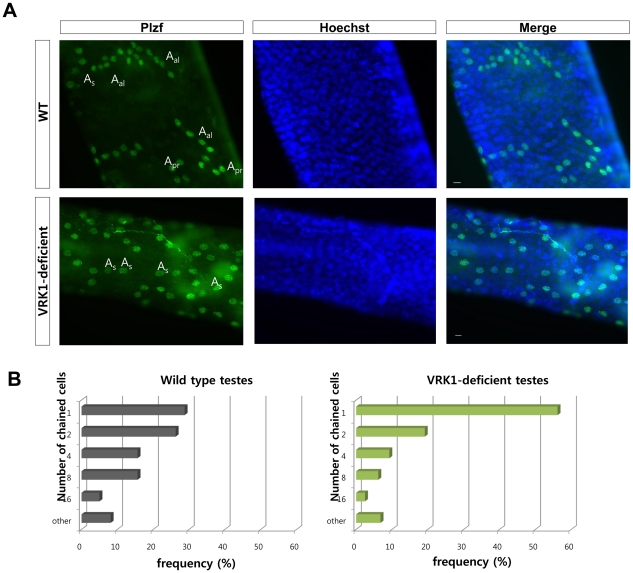
Loss of the chained undifferentiated spermatogonia in VRK1-deficient testes. (A) Five-week-old testes of wild-type and VRK1-deficient mice were examined by whole-mount immunostaining with anti-Plzf antibody. Scale bars, 10 µm. (B) Frequency of the undifferentiated spermatogonia clusters classified by the number of chains of cells. 160 (wild type) or 162 (VRK1-deficient testes) clusters that were clearly identified were counted.

To further analyze the impairment of undifferentiated spermatogonia by VRK1 deficiency, we performed immunostaining of seminiferous tubules with anti-Plzf antibody (Figure 6A and 6B). Plzf is expressed in undifferentiated spermatogonia, that is, in A_s_, A_pr_, and A_al_ [28], [29]. In seminiferous tubules from 5-week-old, wild-type mice, the proportion of A_s_ and A_pr_ were observed with 28.75% and 26.25% frequencies. Compared to wild type, the proportion of each clustered cell type showed significant changes in VRK1-deficient mice. A_s_ cells account for more than 50 percent of the population in VRK1-deficient seminiferous tubules. Whereas A_s_ and A_pr_ were present in 70% of the counted clusters, long-chain undifferentiated spermatogonia were present at lower proportions than wild type. Moreover, Plzf-positive germ cells were seldom detected in seminiferous tubules of 9-week-old mice VRK1-deficient mice, similar to flow cytometry results (Figure 5B and Figure S3). The chain lengths of the remaining undifferentiated spermatogonia were generally short (Figure S3) in VRK1-deficient seminiferous tubules. Type A spermatogonia underwent a series of mitotic divisions into A_pr_, A_al(4)_, A_al(8)_, and A_al(16)_ cells [3]. With these data, we hypothesized that the VRK1 deficiency affects the mitotic division of undifferentiated spermatogonia, resulting in their apparent loss.

## Discussion

This study investigated the functional importance of VRK1 for the maintenance of mammalian spermatogonial stem cells. In view of current genetics analyses, VRK1 is important to the process of gametogenesis. Previous reports have analyzed the function of VRK in *Caenorhabditis elegans* and *Drosophila melanogaster* in which single VRK orthologs exist. siRNA-mediated VRK depletion in *C. elegans* results in early embryonic lethality caused by cell cycle arrest during the earliest cell division [30]. In vrk-1 mutants of *C. elegans* that express truncated VRK-1 proteins, sterility was observed from defects in the cell cycle [31], [32]. NHK1, the VRK1 homolog in *D. melanogaster*, is important for karyosome formation and metaphase arrest during meiosis. Mutations in NHK1 lead to sterility in both male and female flies due to abnormal meiotic spindle formation [33]. NHK1 mutations in oocytes also cause the formation of multiple spindles and increased chromosome condensation during prophase [34]. These reports indicate that VRK1 plays an important role in germ cell development. In contrast to *C. elegans* and *D. melanogaster*, mammalian genomes encode three forms of VRK whose functions are more diverse and complex. Among the VRK families, a hypomorphic mouse model for VRK1 has been recently reported [17]. VRK1 mutant mice generated by the gene trap method showed progressive germ cell loss and resulting infertility. Germ cell nuclear antigen (GCNA), proliferating cell nuclear antigen (PCNA), and c-kit-positive germ cells were all greatly reduced in VRK1-deficient seminiferous tubules. Whereas adult mice showed obvious germ cell defects, testes from prepubertal mice seemed normal. The authors concluded that VRK1 deficiency did not affect the maintenance of spermatogonial stem cells. This conclusion was suggested by counting the number of Plzf-positive cells in the seminiferous tubules during the first wave of spermatogenesis. Previous studies also reported that mice in which essential genes related to the maintenance of spermatogonial stem cells have been knocked out do not frequently show abnormalities in germ cell production during the first wave of spermatogenesis. Few factors other than Plzf [28], [29] and TATA box-binding protein-associated factor 4b (Taf4b) [35] are known to be essential for the maintenance of spermatogonial stem cells. Disruption of the transcriptional repressor Plzf in mice results in a progressive depletion of germ cells, resulting in a phenotype that consists only of Sertoli cells. Similarly, inactivation of Taf4b results in severe germ cell depletion characterized by tubules of only Sertoli cells and male infertility [35]. Although these mice can complete spermatogenesis, leading to the production of normal sperm during the first wave of spermatogenesis, the number of germ cells is reduced, and spermatogonial proliferation is defective at an early age. All of these effects cause infertility in these animals.

In the present research, we analyzed the first wave of spermatogenesis in VRK1-deficient mice and found that the functional defect in spermatogenesis occurred at an early age. Within 2 weeks, the number of actively proliferating spermatogonia was significantly reduced as indicated by cyclin D1 immunostaining (Figure 2B). Moreover, the expression levels of marker genes, including Plzf, POU5f1(Oct-4), POU3f1(Oct-6), Ret, Ngn3, and Lin28, in undifferentiated spermatogonia were markedly downregulated as indicated by microarray analysis and quantitative RT-PCR by day 8 (Figure 3A). Therefore, these results suggest that the impairment of important genes in undifferentiated spermatogonia impede the normal meiotic development of spermatocytes as shown by the GO data (Table 1 and Table S1), indicating that VRK1 plays a crucial role in normal spermatogenesis in neonatal mice.

First, we suppose that VRK1 may be involved in the maintenance of spermatogonial stem cells because VRK1-deficient testes have tubules containing cells at degenerated stages of spermatogenesis or a Sertoli cell-only phenotype as previously reported [17]. These phenotypes typically occur in testes undergoing stem cell loss [3]. Wiebe et al. [13] also expected that degeneration of germ cells is induced by the dysfunctional division of differentiated spermatogonial cells, not by spermatogonial stem cells. However, we revealed that VRK1 deficiency caused a loss of spermatogonial stem cells. We believe that this discrepancy comes from the different methodologies for the quantification of spermatogonial stem cells. Previous reports counted the number of Plzf-positive cells in cross-sections of seminiferous tubules. Although this examination can determine the status of stem cells, it is ambiguous because analyses using cross-sections cannot count all spermatogonial stem cells. Spermatogonial stem cells are a very rare population of all the germ cells, and only a fraction of stem cells can be detected in cross-sectioned tubules. Therefore, most studies quantify spermatogonial stem cells by flow cytometry analysis with antibodies against specific surface marker proteins, which enable the concentration of spermatogonial stem cells for further functional analyses [3], [26]. Our quantification results obtained by flow cytometry provide evidence for the impairment of spermatogonial stem cells in VRK1-deficient mice (Figure 5). Moreover, we found that in 5-week-old VRK1 deficient testes, loss of chained undifferentiated spermatogonia is apparent compared to wild type (Figure 5B). It seems that the proliferation of undifferentiated spermatogonia requires VRK1. Although it is unclear whether VRK1 directly participates in the self-renewal of A_s_ spermatogonial stem cells, a great reduction in the number of spermatogonial stem cells appeared in VRK1-deficient mice. After 8 weeks, VRK1-deficient testes showed a great reduction in spermatogonial stem cells by flow cytometry analysis. In addition, Plzf-positive cells were rarely detected after 9 weeks by whole mount immunohistochemisty of seminiferous tubules (Figure S3). These results indicate that VRK1 is required for the maintenance of spermatogonial stem cells that are regulated by self-renewal.

Spermatogonial stem cells can be regulated by both intrinsic and extrinsic factors; therefore, we asked whether VRK1 directly causes a loss of undifferentiated spermatogonia. Since it is unknown which spermatogonial subpopulation expresses VRK1, we confirmed the expression of VRK1 in undifferentiated spermatogonia by immunocytochemistry after MACS and flow cytometry analysis. We determined that undifferentiated spermatogonia expressing Oct-4 and Plzf but not c-kit also expressed VRK1 (Figure 4). Among the undifferentiated spermatogonia specified as Ep-CAM^+^/α_6_-integrin^+^/c-kit^−^, approximately 66% of cells expressed VRK1 (Figure 4E). These data suggest the existence of a specific subpopulation in undifferentiated spermatogonia that expresses VRK1. Another possibility is that the level of VRK1 changes during the phases of the cell cycle. As VRK1 is highly expressed during the G2-M phases of the cell cycle in somatic cells, the expression levels of VRK1 might also oscillate during the cell cycle in proliferating spermatogonia. This idea might contribute to the detection of undifferentiated spermatogonia that are in the G2-M phase. Together, we suggest that VRK1 is a crucial intrinsic factor for the maintenance of spermatogonial stem cells.

Here, we suggest VRK1 mainly exists in spermatogonia, but not in Sertoli cells. VRK1 protein was detected in spermatogonia with anti-mouse VRK1 antibody but not in Sertoli cells (Figure 1C). In the previous study, GT12/GT12 (a gene-trap vector insertion within intron 12 of *Vrk1*) tubule was stained with x-gal [17]. Although this data confirmed the promoter activity of VRK1in spermatogonia and Sertoli cells, it does not determine whether VRK1 protein exists or not. Proteolysis is a crucial mechanism for the regulation of VRK1 [9]. But mutant VRK1 deleted in exon 13–15 may not be normally regulated because C-terminal region of VRK1 has been suggested as a regulatory domain interacting with other proteins [36]. Therefore, strong staining for beta-galactosidase in Sertoli cells may be due to the accumulation of mutant VRK1 protein.

Although VRK1 is not detected in Sertoli cells by immunohistochemistry, undetectable amount of VRK1 protein might exist. In this case, it cannot be overlooked that an impairment in the maintenance of undifferentiated spermatogonia can be caused by a malfunction of the stem cell niche supported by Sertoli cells. Wiebe et al. showed that expression levels of the kit ligand were unaltered in VRK1-deficient testes, suggesting that VRK1-deficient Sertoli cells retain their ability to secrete important paracrine factors [17]. To prove that VRK1 is an intrinsic factor for the maintenance of undifferentiated spermatogonia, repopulation assays using transplantation of VRK1-deficient germ cell are needed.

Overall, our present study showed that the proliferative capability of undifferentiated spermatogonia, including spermatogonial stem cells, is compromised at an early postnatal age by VRK1-deficiency. Furthermore, in view of the specific biochemical role of VRK1 in the progression of the cell cycle, these data implicate VRK1 in the mitotic regulation of spermatogonial stem cell maintenance. To elucidate the molecular mechanisms of VRK1 in male germ cells, further investigation is expected to clarify this important issue.

## Materials and Methods

### Ethics Statement

Approval of the study protocol was obtained from the Pohang University of Science and Technology Institutional Animal Care and Use Committee (approval ID: LIFE 012). All animal experiments carried out according to the provisions of the Animal Welfare Act, PHS Animal Welfare Policy, and the principles of the NIH Guide for the Care and Use of Laboratory Animals. All mouse lines were maintained under specific pathogen-free conditions at the POSTECH animal facility under institutional guidelines.

### Mice

The gene-trapped ES cell line RRR178 was obtained from BayGenomics. This cell line was generated using a gene trap protocol with the pGT1lxf construct, which contains the intron from *engrailed 2* upstream of a gene encoding β-galactosidase/neomycin resistance, β-geo (see http://www.genetrap.org). The ES cell clone was injected into a C57BL/6 blastocyst according to standard procedures. Male chimeras were bred with C57BL/6 mice to create animals with a germ-line transmission of the mutant allele. Heterozygous mice were backcrossed a minimum of six generations with C57BL/6 mice prior to the study. Genotyping was performed by PCR and Southern blot using DNA extracted from mouse tails. Insertion of the β-geo cassette was verified by PCR (β-geo primer sense, 5′-ATCGCAGATCTGGACTCTAGAGGATCC-3′ and antisense, 5′-ATGCGCTCAGGTCAAATTCAGACGGCAA-3′ and Int4 primer sense, 5′-ATCGCAGATCTGGACTCTAGAGGATCC-3′ and antisense, 5′-GGAGAAACTTTGTACAGCTTCGTT-3′, 5′-AAGGAATCTTGGTTAGCTTTCAGA-3′).

### Preparation of the anti-mouse VRK1 antibody

Mouse VRK1 antisera were generated in rabbits using recombinant mouse VRK1 (accession number NM_011705.3) as the immunogen. Approximately 1 mg recombinant mouse VRK1 was used to immunize rabbits with complete Freund's adjuvant by subcutaneous injection. Two weeks after the first immunization, the rabbits were boosted with incomplete adjuvant. Rabbits were boosted once more with only recombinant protein weeks after the second immunization. Rabbit serum was collected and subjected to affinity purification using a HiTrap Protein G column (GE Healthcare).

### Histology and β-gal histochemistry

For histological analyses, testes were fixed in cold 4% paraformaldehyde, processed into paraffin sections, and stained with hematoxylin and eosin. Histochemical analysis of β-galactosidase reporter activity was performed with frozen sections following the standard method.

### In situ hybridization

A 349-base pair fragment of VRK1 cDNA was obtained by RT-PCR with the use of the oligonucleotides VRK1-349 (f), 5′-TGGAAAAAGTTACAGGTTTATGATAATG -3′, and VRK1-349 (r), 5′-GTAGTTTCACAGACTCCATGTACTTAGC-3′. Synthesis of both anti-sense and sense probes was performed followed by cloning into the pGEM-easy vector. Cryostat sections were hybridized with probes labeled with digoxygenin. Samples were incubated with anti-DIG POD antibody (Roche) and detected using Cy3 Tyramide Signal Amplification (TSA, PerkinElmer, Waltham, MA).

### Immunohistochemistry and immunocytochemistry

Sorted cells were attached to a poly-D-lysine glass slide for immunocytochemistry and then stained with antibodies against VRK1 and Oct4 (sc-5279, Santa Cruz Biotechnology, Santa Cruz, CA) or Plzf (OP128, Calbiochem, San Diego, CA). For immunohistochemistry, testes were fixed in 4% paraformaldehyde, dehydrated using an ethanol series, and embedded in paraffin. Sectioned tissues (5 µm) were dehydrated and stained with antibodies.

### Flow Cytometry

To analyze undifferentiated spermatogonial cell populations, spermatogonial cell populations were enriched using magnetic-activated cell sorting (MACS) (Miltenyi Biotech, Bergisch Gladbach, Germany). Dissociated testicular cells (1×10^7^) were incubated with anti-Ep-CAM antibody followed by anti-rat IgG secondary antibody conjugated to microbeads. Ep-CAM^+^ cells were resuspended in PBS containing 1% FBS and then stained with APC-conjugated anti-c-kit, R-phycoerythrin (PE)-conjugated anti-α6-integrin, and biotin-conjugated anti-Thy-1.2 antibodies prior to flow cytometry analysis. PerCP-conjugated streptavidin was used as a second step to detect Thy-1.2. To identify VRK1 expression, Ep-CAM positive and negative cells were labeled with fluorescein di-D-galactopyranoside (FDG; Invitrogen) per the manufacturer's protocol. For the staining with FDG, stock solution (20 mM) was diluted 1∶10 with DW and prewarmed at 37°C. This solution was mixed with same volume of PBS and incubated with cells for 1 min at 37°C. After incubation, cells were washed with 1 ml of cold PBS. Cells were further stained with (PE)-conjugated anti-α6-integrin antibody for identifying undifferentiated spermatogonial population. All antibodies were purchased from BD Biosciences (San Jose, CA). Flow cytometry was performed on a FACSCalibur (BD Biosciences).

### Quantitative RT-PCR

Total RNA samples from day8 wild type and VRK1-deficient mice (n = 4, each) were prepared from intact testicular cells using Trizol (Invitrogen, Carlsbad, CA) and then reverse transcribed using the ImProm-II Reverse Transcription System (Promega). Quantitative PCR was performed on a MyIQ single time, real-time detector system (Bio-Rad, Hercules, CA) using the SYBR Green PCR mixture (TAKARA BIO INC.) as described previously [37]. β-actin expression was used to normalize transcript levels. Relative values of transcripts were derived using the equation, 2^−ΔΔ*Ct*^, where Δ*Ct* is equal to the difference in threshold cycles. PCR primer sequences are listed in supplemental Table S2. Error bars were calculated as the range in fold change of VRK1-deficient transcript levels (mean ± standard deviation) relative to the mean of wild type transcript.

### Whole-mount immunofluorescence staining

Testes were removed from their capsules and placed in cold PBS. Tubules were dissociated from each other and removed from interstitial material with 1 mg/ml collagenase IV. After fixation in PBS containing 4% paraformaldehyde, the samples were incubated in blocking solution. Spermatogonial stem cells were stained with a Plzf antibody (Calbiochem, OP128) followed by staining with an Alexa Fluor 488-conjugated goat anti-mouse antibody. After washing in PBS, tubules were stained for 10 min with Hoechst 33342 in PBS and mounted in Vectashield mounting medium (Vector Laboratories, Burlingame, CA).

### DNA microarray analysis

Global gene expression analysis was conducted using Agilent Whole Genome Mouse Microarrays and One-Color Quick Amp Labeling Kit (Agilent Technologies, Inc., Santa Clara, CA) according to the manufacturer's protocol. Briefly, 0.5 µg of total RNA was reverse transcribed using T7 promoter primers and MMLV reverse transcriptase. cRNA was amplified by T7 RNA polymerase and labeled with Cy-3 dye, followed by column purification provided in the RNeasy Mini Kit (Qiagen, Inc., Chatsworth, CA). Labeled samples were hybridized with probes on the microarrays at 65°C for 17 hours, and the microarrays were scanned using an Agilent Microarray Scanner after washing out unbound samples. Microarray expression data were extracted and analyzed using the Agilent Feature Extraction Software and GeneSpring GX respectively. Fold changes were calculated as a ratio between signal averages of four biological replicates of wild type and VRK1-deficient mice with statistical significance given by the Student's t-test (cutoff p-value = 0.05), and these significantly changed genes were used for gene ontology analysis. The raw data have been deposited in NCBI's Gene Expression Omnibus and are accessible through GEO Series accession number GSE22674.

## Supporting Information

Figure S1 **Characterization of VRK1‐deficient mice.** (A) Schematic representation of the wild type and mutant (VRK1‐deficient) gene locus of VRK1. The insertion site, corresponding coding exons (light gray) and noncoding exons (open box), and the β‐geo cassette (yellow) are shown. Thin (noncoding) and thick (coding sequences) lines under exons represent the expected transcripts derived from the wild type and VRK1‐deficient alleles. β‐geo indicates the bacterial β‐galactosidase fused to the neomycin resistance gene. Primers for genotyping are represented by arrows. Red box indicates the region for the Southern blot probe. (B) PCR analysis of genomic DNA (gDNA) isolated from three littermates produced by heterozygotes crosses. (Upper panel) PCR product amplified with Int4 primers showing a 600‐bp band from gDNA of wild type and heterozygote mice. (Lower panel) PCR product amplified with β‐geo primers showing a 550‐bp band from gDNA of wild type and VRK1‐deficient mice. (C) Southern blot analysis of genomic DNA. Probing of BamHI‐digested DNA revealed 7.5‐kb and 5.5‐kb fragments for the wild type and VRK1‐deficient alleles, respectively. (D) mRNA levels of VRK1 in different tissues harvested from wild type and VRK1‐deficient mice. Semi‐quantitative PCR was performed with primers that amplify Exon2 to Exon4. β‐actin was used as a loading control.
(TIF)

Figure S2 **The validation of mouse VRK1 antibody.** (A) Immunoblot of endogenous mouse VRK1 in spleen and recombinant mouse VRK1 protein with anti‐mVRK1 antibody. (B) Expression pattern of VRK1 in wild type and VRK1‐deficient mouse organs. Lamin B was used as a loading control.
(TIF)

Figure S3 **Loss of undifferentiated spermatogonia in VRK1‐deficient testes.** nine‐week‐old testes of wild‐type and VRK1‐deficient mice were examined by whole‐mount immunostaining with anti‐Plzf antibody. Scale bars, 10 µm.
(TIF)

Table S1 Gene ontology terms of the genes with a significant change in the expression level in VRK1‐deficient mice.
(TIF)

Table S2 Primers for qRT‐PCR.
(TIF)
