# Supplementary figures and images for: Vaccinia-Related Kinase 1 Is Required for the Maintenance of Undifferentiated Spermatogonia in Mouse Male Germ Cells
Source: PLoS One. 2010 Dec 13;5(12):e15254. doi: 10.1371/journal.pone.0015254 (PMC3001494; doi:10.1371/journal.pone.0015254)

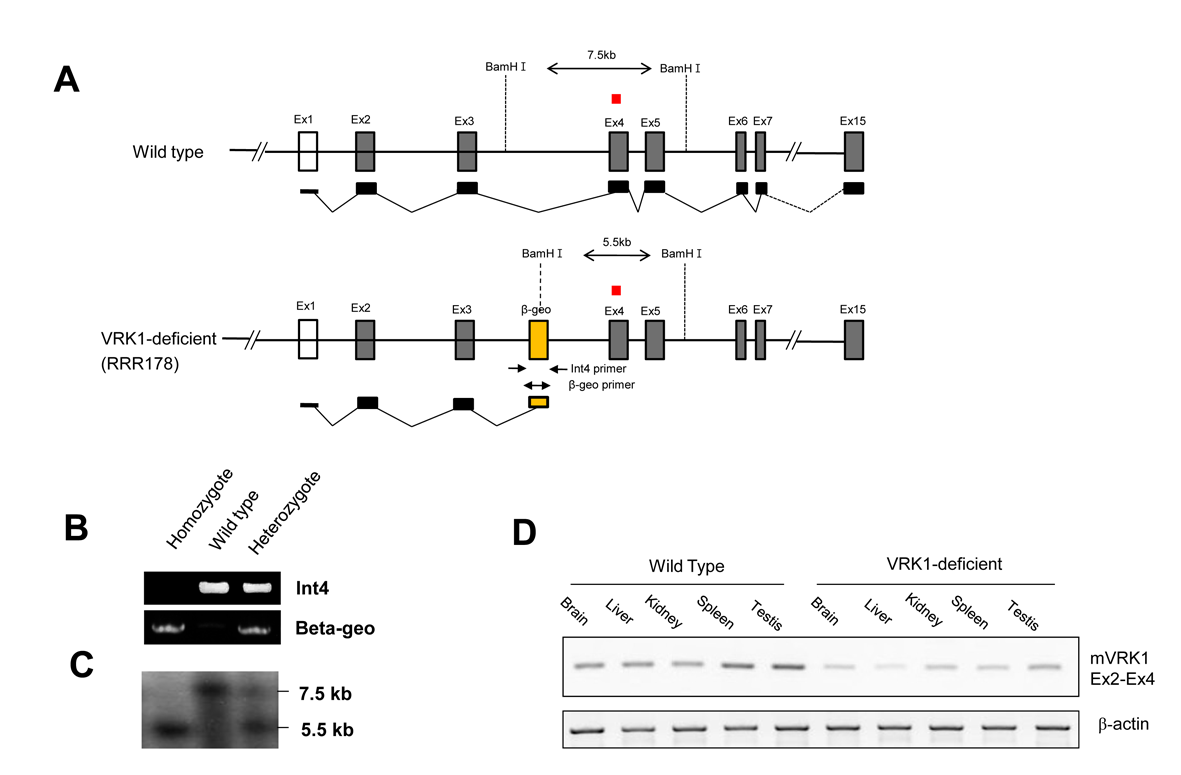

Supplement: Figure S1 — Characterization of VRK1‐deficient mice. (A) Schematic representation of the wild type and mutant (VRK1‐deficient) gene locus of VRK1. The insertion site, corresponding coding exons (light gray) and noncoding exons (open box), and the β‐geo cassette (yellow) are shown. Thin (noncoding) and thick (coding sequences) lines under exons represent the expected transcripts derived from the wild type and VRK1‐deficient alleles. β‐geo indicates the bacterial β‐galactosidase fused to the neomycin resistance gene. Primers for genotyping are represented by arrows. Red box indicates the region for the Southern blot probe. (B) PCR analysis of genomic DNA (gDNA) isolated from three littermates produced by heterozygotes crosses. (Upper panel) PCR product amplified with Int4 primers showing a 600‐bp band from gDNA of wild type and heterozygote mice. (Lower panel) PCR product amplified with β‐geo primers showing a 550‐bp band from gDNA of wild type and VRK1‐deficient mice. (C) Southern blot analysis of genomic DNA. Probing of BamHI‐digested DNA revealed 7.5‐kb and 5.5‐kb fragments for the wild type and VRK1‐deficient alleles, respectively. (D) mRNA levels of VRK1 in different tissues harvested from wild type and VRK1‐deficient mice. Semi‐quantitative PCR was performed with primers that amplify Exon2 to Exon4. β‐actin was used as a loading control. (TIF) [file pone.0015254.s001.tif]

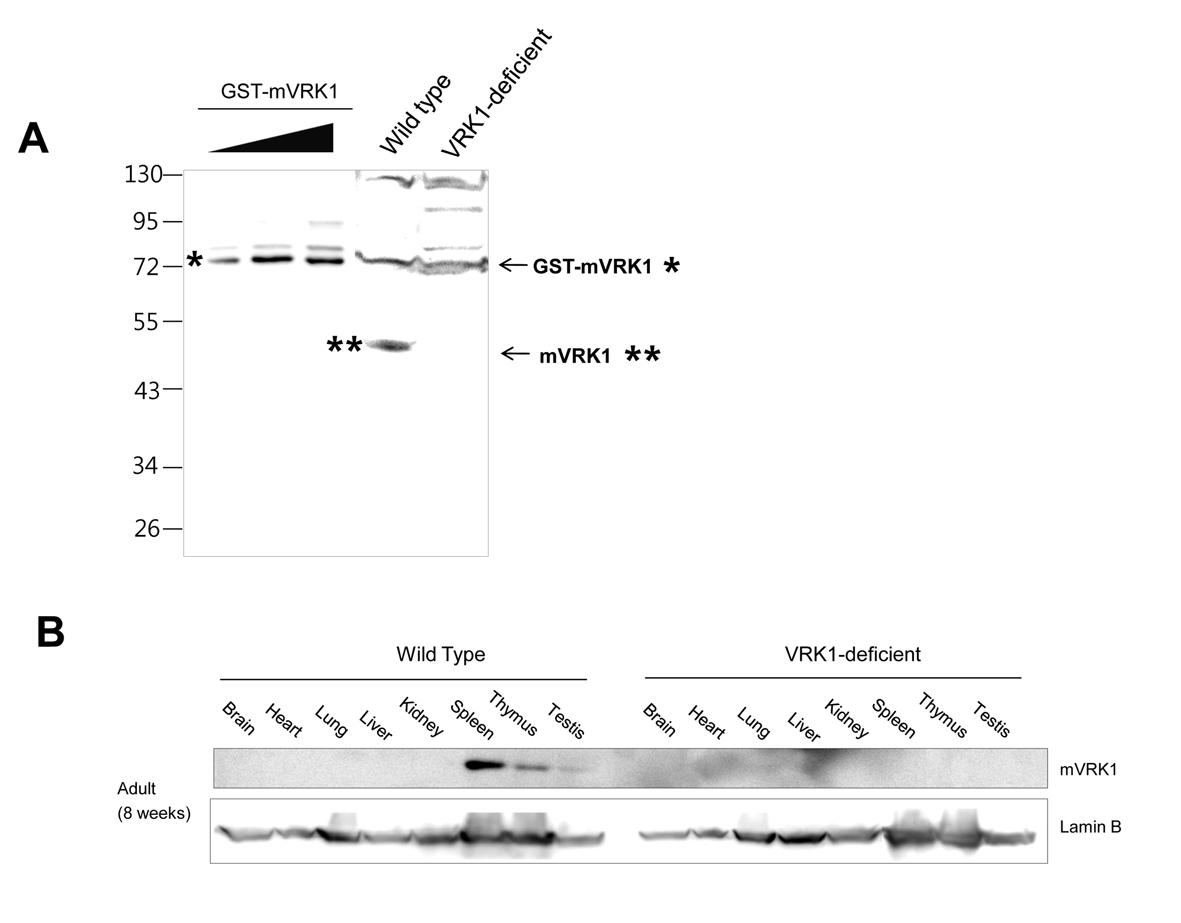

Supplement: Figure S2 — The validation of mouse VRK1 antibody. (A) Immunoblot of endogenous mouse VRK1 in spleen and recombinant mouse VRK1 protein with anti‐mVRK1 antibody. (B) Expression pattern of VRK1 in wild type and VRK1‐deficient mouse organs. Lamin B was used as a loading control. (TIF) [file pone.0015254.s002.tif]

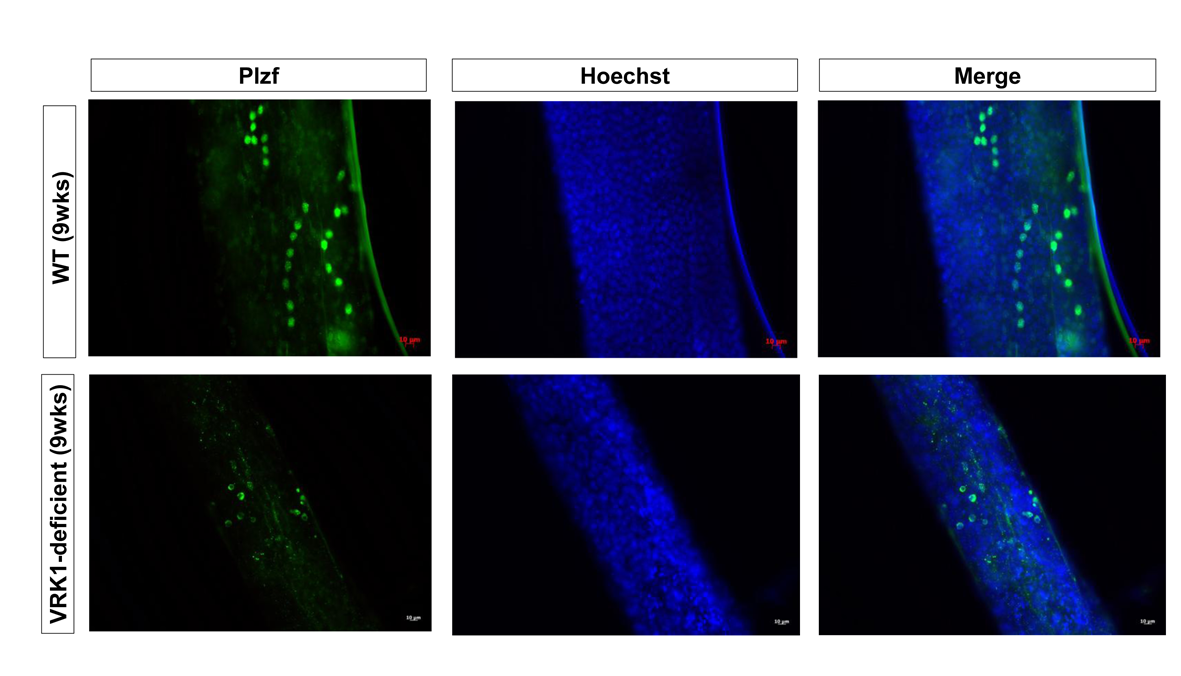

Supplement: Figure S3 — Loss of undifferentiated spermatogonia in VRK1‐deficient testes. nine‐week‐old testes of wild‐type and VRK1‐deficient mice were examined by whole‐mount immunostaining with anti‐Plzf antibody. Scale bars, 10 µm. (TIF) [file pone.0015254.s003.tif]

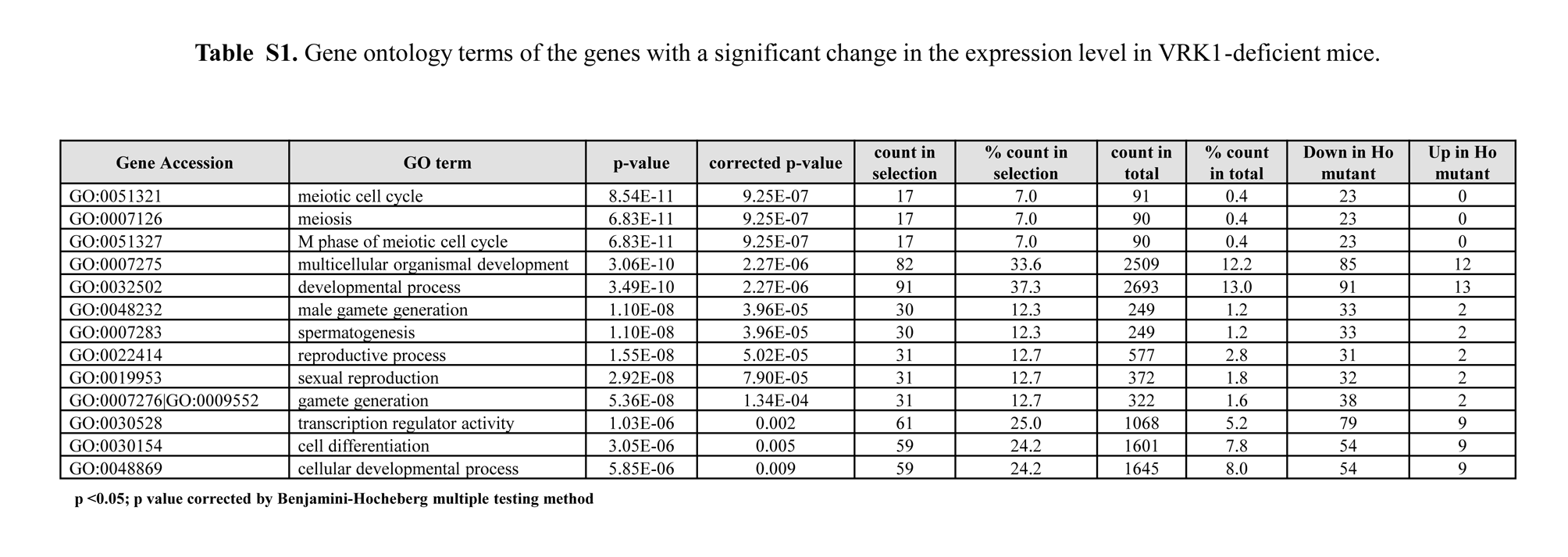

Supplement: Table S1 — Gene ontology terms of the genes with a significant change in the expression level in VRK1‐deficient mice. (TIF) [file pone.0015254.s004.tif]

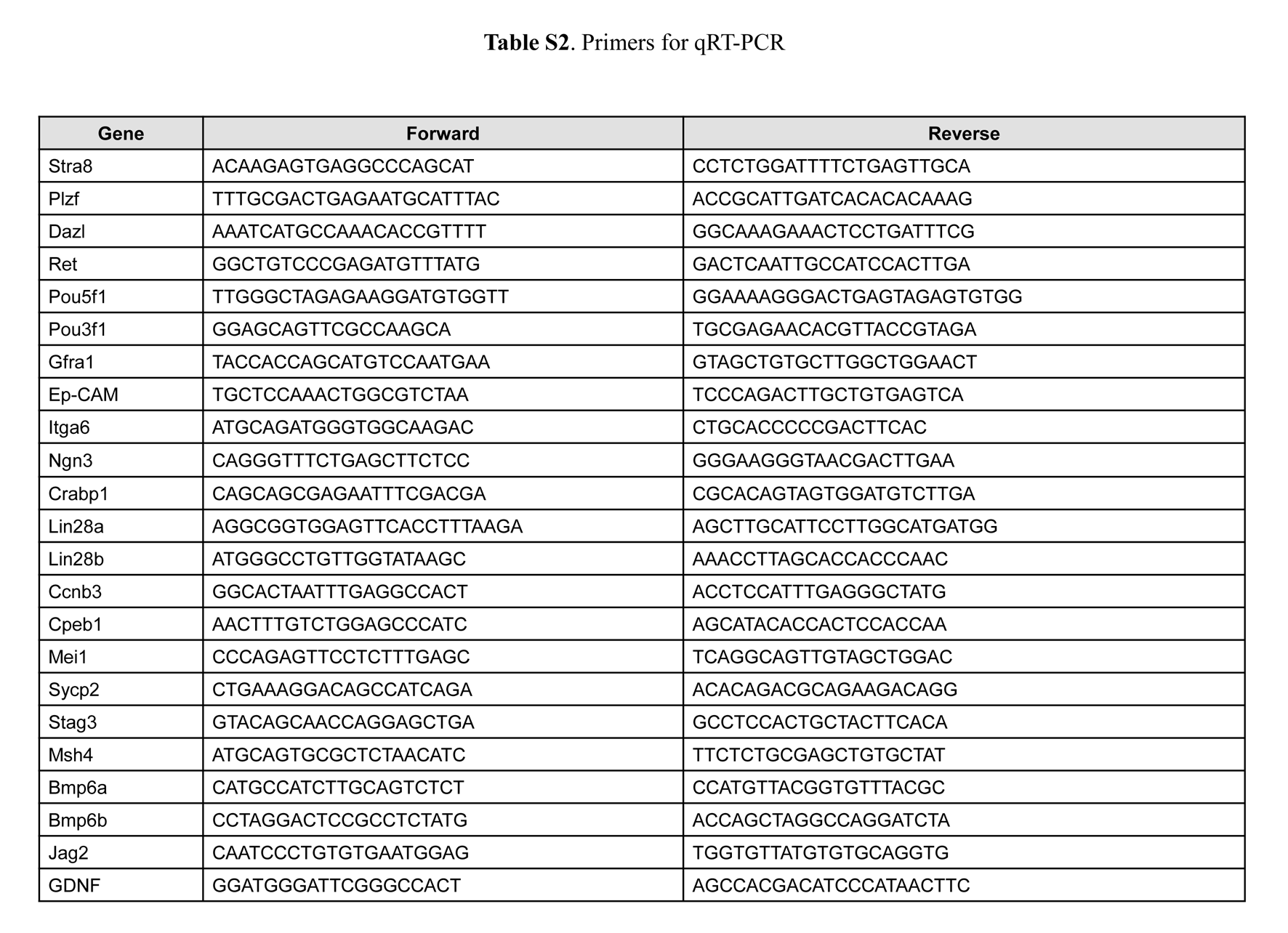

Supplement: Table S2 — Primers for qRT‐PCR. (TIF) [file pone.0015254.s005.tif]
